# Exploring the frequency of a *TP53* polyadenylation signal variant in tumor DNA from patients diagnosed with lung adenocarcinomas, sarcomas and uterine leiomyomas

**DOI:** 10.1590/1678-4685-GMB-2023-0133

**Published:** 2024-01-19

**Authors:** Igor Araujo Vieira, Guilherme Danielski Viola, Eduarda Heidrich Pezzi, Thayne Woycinck Kowalski, Bruna Vieira Fernandes, Tiago Finger Andreis, Natascha Bom, Giulianna Sonnenstrahl, Yasminne Marinho de Araújo Rocha, Bruno da Silveira Corrêa, Luiza Mezzomo Donatti, Gabriela dos Santos Sant’Anna, Helena von Eye Corleta, Ilma Simoni Brum, Clévia Rosset, Fernanda Sales Luiz Vianna, Gabriel S. Macedo, Edenir Inez Palmero, Patricia Ashton-Prolla

**Affiliations:** 1 Universidade Federal do Rio Grande do Sul, Programa de Pós-Graduação em Genética e Biologia Molecular, Porto Alegre, RS, Brazil.; 2 Hospital de Clínicas de Porto Alegre (HCPA), Centro de Pesquisa Experimental, Laboratório de Medicina Genômica, Porto Alegre, RS, Brazil.; 3 Universidade do Vale do Rio dos Sinos (UNISINOS), Escola de Saúde, São Leopoldo, RS, Brazil.; 4 Universidade Federal do Rio Grande do Sul (UFRGS), Laboratório de Genética Médica e Populacional, Porto Alegre, RS, Brazil.; 5 Instituto Nacional de Genética Médica Populacional (INAGEMP), Porto Alegre, RS, Brazil.; 6 Hospital de Clínicas de Porto Alegre (HCPA), Serviço de Genética Médica, Sistema Nacional de Informações sobre Agentes Teratogênicos (SIAT), Porto Alegre, RS, Brazil.; 7 Complexo de Ensino Superior de Cachoeirinha (CESUCA), Cachoeirinha, RS, Brazil.; 8 Universidade do Vale do Rio dos Sinos (UNISINOS), Curso de Graduação em Biomedicina, São Leopoldo, RS, Brazil.; 9 Universidade Federal do Rio Grande do Sul, Instituto de Ciências Básicas da Saúde, Departamento de Fisiologia, Laboratório de Biologia Molecular Endócrino e Tumoral, Porto Alegre, RS, Brazil.; 10 Universidade Federal do Rio Grande do Sul, Programa de Pós-Graduação em Ciências Biológicas: Fisiologia, Porto Alegre, RS, Brazil.; 11 Hospital de Clínicas de Porto Alegre (HCPA), Serviço de Ginecologia e Obstetrícia, Porto Alegre, RS, Brazil.; 12 Universidade Federal do Rio Grande do Sul, Faculdade de Medicina, Departamento de Ginecologia e Obstetrícia, Porto Alegre, RS, Brazil.; 13 Universidade Federal do Rio Grande do Sul (UFRGS), Programa de Pós-Graduação em Ciências Médicas: Medicina (PPGCM), Porto Alegre, RS, Brazil.; 14 Hospital de Clínicas de Porto Alegre (HCPA), Unidade de Pesquisa Laboratorial (UPL), Porto Alegre, RS, Brazil.; 15 Universidade Federal do Rio Grande do Sul, Departamento de Genética, Laboratório de Imunobiologia e Imunogenética, Porto Alegre, RS, Brazil.; 16 Hospital de Clínicas de Porto Alegre (HCPA), Programa de Medicina Personalizada, Porto Alegre, RS, Brazil.; 17 Instituto Nacional de Câncer (INCA), Departamento de Genética, Rio de Janeiro, RJ, Brazil.; 18 Hospital de Câncer de Barretos, Centro de Pesquisa em Oncologia Molecular, Barretos, SP, Brazil.; 19 Hospital de Clínicas de Porto Alegre, Serviço de Genética Médica, Porto Alegre, RS, Brazil.

**Keywords:** rs78378222, non-coding variant, 3’ untranslated region, *TP53* gene, somatic analyses

## Abstract

The *TP53* 3’UTR variant rs78378222 A>C has been detected in different tumor types as a somatic alteration that reduces p53 expression through modification of polyadenylation and miRNA regulation. Its prevalence is not yet known in all tumors. Herein, we examine tumor tissue prevalence of rs7837822 in Brazilian cohorts of patients from south and southeast regions diagnosed with lung adenocarcinoma (LUAD, n=586), sarcoma (SARC, n=188) and uterine leiomyoma (ULM, n=41). The minor allele (C) was identified in heterozygosity in 6/586 LUAD tumors (prevalence = 1.02 %) and none of the SARC and ULM samples. Additionally, next generation sequencing analysis revealed that all variant-positive tumors (n=4) with sample availability had additional pathogenic or likely pathogenic somatic variants in the *TP53* coding regions. Among them, 3/4 (75 %) had the same pathogenic or likely pathogenic sequence variant (allele frequency <0.05 in tumor DNA) namely c.751A>C (p.Ile251Leu). Our results indicate a low somatic prevalence of rs78378222 in LUAD, ULM and SARC tumors from Brazilian patients, which suggests that no further analysis of this variant in the specific studied regions of Brazil is warranted. However, these findings should not exclude tumor molecular testing of this *TP53* 3’UTR functional variant for different populations.

## Introduction

Somatic *TP53* sequence variants are the most frequent alterations in human cancers. Although most studies focusing on molecular detection of these variants analyze exonic regions and exon-intron boundaries, *TP53* sequence variants in 5’ and 3’ untranslated regions (UTR) have been recently described as tumor-promoting alterations (Stacey *et al*., 2011; Li *et al*., 2013; Diederichs *et al*., 2016; Deng *et al*., 2019). The 5’- and 3’- UTRs are highly conserved elements. The 5’ UTR is critical for ribosome recruitment to the mRNA, playing a major role in the control of translation (Hinnebusch *et al*., 2016). Of particular interest, 3’ UTRs are often proposed as binding sites for microRNAs (miRNAs) and harbor polyadenylation regulatory sequences that govern mRNA stability, localization, and protein translation efficiency (Matoulkova *et al*., 2012; Jardin and Coiffier, 2013; Erson-Bensan, 2020). Thus, any sequence variations occurring in both UTRs may exert a significant influence on protein expression. In this context, Li *et al.* (2013) first analyzed both *TP53* UTRs and showed that somatic alterations occurred frequently in these regions and that they had prognostic value in a large cohort of tumor specimens from patients with a subtype of lymphoma (Li *et al.*, 2013). 

The detection of a rare variant (rs78378222, NM_000546.5: c.*1175A>C) in the sole polyadenylation signal (PAS) sequence of *TP53* initially associated with basal cell carcinoma has brought attention to this genomic region (Stacey *et al*., 2011; Wang *et al*., 2016). Ten years after its first description, the rs78378222[C] allele has been described as a risk allele for development to several tumors (Zhou *et al*., 2012; Enciso-Mora *et al*., 2013; Diskin *et al*., 2014; Rafnar *et al*., 2018; Deng *et al*., 2019; Di Giovannantonio *et al*., 2021). Recently, *in vitro* and *in vivo* analyses showed further tumor-promoting mechanisms associated with this variant. In addition to the disruption of the PAS sequence, it creates and alters miRNA binding sites in the *TP53* 3’UTR (Deng *et al.*, 2019; Zhang *et al.*, 2021). 

In previous studies based on germline analyses, the rs78378222[C] minor allele was significantly associated with increased risk for uterine leiomyoma (ULM) and soft-tissue sarcomas (SARC) in European and Chinese populations, respectively (Rafnar *et al*., 2018; Deng *et al*., 2019). In contrast, this variant was not previously associated with the occurrence of lung tumors in general (undefined histological subtypes) in a germline study conducted in the USA population (Guan *et al*., 2013). So far, only one previous study investigated the germline frequency of this variant in a sample of individuals from the south and southeast regions of Brazil, including a control group, breast cancer and Li-Fraumeni syndrome-affected patients (Macedo *et al*., 2016). Considering the increased risk to develop ULM and SARC in rs78378222[C] germline carriers, as well as the lack of studies focusing on lung adenocarcinoma (LUAD), new studies exploring the frequency of this variant in the somatic context for these tumor types and in different populations are required. Hence, the aim of this study was to determine the somatic prevalence of the *TP53* rs7837822 (A>C) variant in a group of LUAD tumors, SARC and ULM cases from Southern and Southeast Brazil. We also characterized the clinical and molecular features associated with variant-positive patients.

## Subjects and Methods

### Study subjects, sample types and ethical aspects

Patients over 18 years of age diagnosed with lung adenocarcinoma (LUAD, n= 586), uterine leiomyoma (ULM, n=41) and sarcomas (SARC, n=188) were recruited from tertiary care public and private hospitals and clinics located in four states of the southern and southeastern regions of Brazil. Patients were not selected if they had previous cancer diagnosis or cancer family history. Pathology analyses confirmed typical adenocarcinoma histology in all lung cancer cases.

Formalin‐fixed, paraffin‐embedded (FFPE) LUAD tissues were obtained from a case series originally reported in a previous retrospective study that conducted somatic testing of *EGFR*, *KRAS*, *BRAF*, and *NRAS* genes in the samples (Andreis *et al*., 2019). The same LUAD sampling used here was included in a recent work of our group analyzing the prevalence of a *TP53* founder variant (Vieira *et al*., 2021). Ethnic ancestry data were not available for the LUAD patients due to the retrospective design for recruitment.

Sarcomas (SARC) tissues were obtained from patients recruited in Hospital de Câncer de Barretos (currently known as Hospital de Amor) between 2008 and 2016, as previously reported in the original study (Volc *et al*., 2020). Briefly, frozen samples were selected from the Institutional Biobank, macrodissected and revised by a board of pathologists who decided the best area to be analyzed (areas with tumor content higher than 60 % and necrosis lower than 20 %). SARC-affected individuals were predominantly self-declared white (60 %) and mostly Caucasians/Euro-descendants in genetic ancestry analysis (Volc *et al.*, 2020).

Uterine leiomyoma (ULM) frozen tumor (uterine fibroids) and tumor-adjacent normal tissue (myometrium) paired samples from ULM-affected women were derived from one prospective cohort study conducted at the Gynecology and Obstetrics Service of HCPA. ULM samples were predominantly from Caucasians/Euro-descendant individuals.

All methods were carried out in accordance with relevant guidelines and regulations. Before the beginning of the LUAD retrospective study (Andreis *et al*., 2019), age at LUAD diagnosis, *EGFR*/*KRAS*/*BRAF*/*NRAS* status, and histological subtype (when available) were annotated, and samples were further de-identified. All genetic analyses were previously approved by the Research Ethics Committee of HCPA, Brazil (No. 2018-0099 for LUAD, No. 2018-0517 for ULM) and registered under the Certificate of Presentation for Ethical Appreciation (CAAE No. 83557418.5.0000.5327 for LUAD, and No. 93970518.0.0000.5327 for ULM). Lastly, molecular testing involving *TP53* gene in SARC tumors was previously approved by the local institutional ethical committee (Hospital de Câncer de Barretos, approval number 866/2014). 

### Molecular analyses

For LUAD FFPE samples containing a high percentage of tumor cells, DNA extraction was performed using the ReliaPrep FFPE gDNA Miniprep System (Promega) according to the manufacturer’s instructions. For SARC frozen samples, genomic DNA was extracted from tumor tissue using a DNA Blood and Tissue kit (Qiagen) according to the manufacturer’s recommendations. For ULM-affected and unaffected frozen samples, DNA extraction was carried out using GenElute™ Mammalian Genomic DNA Miniprep Kit (Sigma-Aldrich) according to the manufacturer’s recommendations.

*TP53* rs7837822 genotyping was performed in duplicate by real-time PCR using fluorescent allele-specific TaqMan^®^ probes (reference and catalog numbers C_102214636_10 and 4351379, respectively), according to the Applied Biosystems^®^ standard protocols (Applied Biosystems, Carlsbad, USA). 

Detection of additional *TP53* sequence variants in rs78378222[C]-positive LUAD tumors was performed by next generation sequencing (NGS) analyses of the *TP53* entire coding region (exons 2-11) and 70 bp exon-intron junctions (not including the position of the studied variant at 3’UTR) using a custom panel (Thermo Fisher Scientific, Waltham, MA; reference number TP53.20140108.designed) on the Ion Torrent PGM platform (Thermo Fisher Scientific). Amplicon library was prepared using the Ion AmpliSeq^TM^ Library Kit 2.0 (Thermo Fisher Scientific) and then the PCR products were sequenced on the Ion GeneStudio S5 system (Ion Torrent Systems Inc., Gilford, NH). Data were analyzed on the bioinformatics platform Ion Reporter version 5.0 with a minimum coverage of 100X by amplicon. Sequence NM_000546.5 was used as a wild-type (WT) *TP53* reference. The NGS analyses were performed using research-use-only reagents with internal validation.

### Statistical analyses

Genotype and allele frequencies were estimated by simple counting. Clinical and molecular features of patients were assessed using descriptive statistics. Considering the low number of individuals harboring the variant allele found in our study and limitations in clinical data availability, it was not possible to perform any meaningful statistical test in our comparisons between groups of variant carriers and non-carriers (see more in Results section). SPSS^®^ version 18 (SPSS^®^ Inc., Chicago, IL, USA) was used for data handling and for all descriptive analyses.

## Results

**Figure 1 -:**
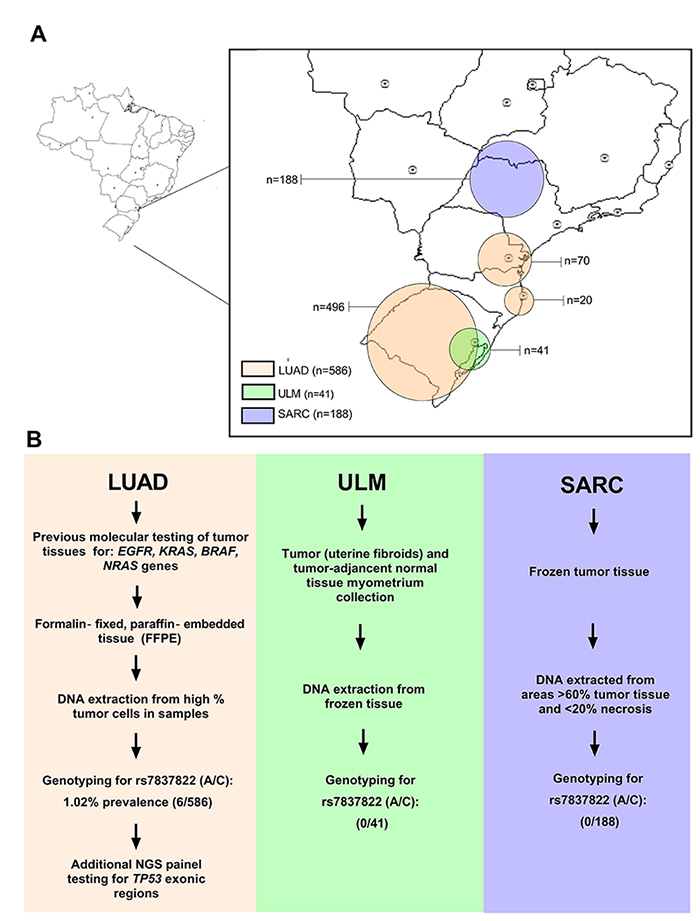
A) Geographic distribution of samples’ origin from Brazilian patients included in the present study. B) Study workflow according to the different tumor types analyzed.

Clinical samples obtained from a total of 815 individuals diagnosed with lung adenocarcinoma (LUAD), sarcoma (SARC) and uterine leiomyoma (ULM) were included in this study. Figure 1 summarizes the workflow of the study, while general clinical data according to the study groups are summarized in Table 1. The mean and median age at tumor/condition diagnosis was concentrated in the sixth decade of life in LUAD patients, and in the fourth decade in SARC and ULM patients. Other specific clinical features of each tumor/condition are presented in Table S1, where most ULM cases (51.5 %) presented uterine location classified as intramural. Sarcomas were classified as soft tissue, bone or leiomyosarcoma types.

**Table 1 -:** General clinical features from patients included in this study according to the tumor type.

Clinical features	Lung adenocarcinoma, LUAD (n=586)	Uterine leiomyoma, ULM (n=41)	Sarcoma, SARC (n=188)
Gender, N ( %)			
Male	271 (46.2)	NAP	111 (59.1)
Female	315 (53.8)	41 (100)	77 (40.9)
Self-identified ethnicity,			
N ( %)^a^		34 (82.9)^a^	
White	NAV	24 (70.6)	NAV
Black	NAV	10 (29.4)	NAV
Age at diagnosis			
Mean, years (±SD)	66.1 (11.9)	43.7 (5)	40.85 (21.12)
Median, years (IR)	67 (16)	44 (6.5)	42 (36)

SD, standard deviation; IR, interquartile range; NAP, not applicable; NAV, not available due to the retrospective study design for recruitment of LUAD and SARC specimens, hindering the inquiry about ethnic ancestry.

^a^ The percentage was calculated over the total number of genotyped samples for each study group, and over the number of cases for which the specified clinical data was available.

Allele and genotype frequencies of *TP53* rs78378222 in each group of analysis are shown in Table 2. Among 586 LUAD tumor specimens, we detected 6 tumors with the PAS variant in heterozygosity. Therefore, the somatic frequencies of the rs78378222[A/C] genotype and C allele were 6/586 (1.02 %) and 6/1172 (0.51 %), respectively. Most of the heterozygotes were diagnosed with cancer late in life (>70 years; 4/6, 66,7 %), which is expected for cancer occurrence in the general population. Only one of the six heterozygotes identified had a cancer diagnosis at an early age: a LUAD-affected female diagnosed at age 44, with a somatic activating *EGFR* alteration in the tumor (Table 3). In addition, two LUAD tumors harboring the rs78378222[C] allele exhibited the somatic oncogenic driver variant *KRAS* p.(Gly12Cys). The other three LUAD variant-positive specimens had no identifiable somatic *EGFR*, *KRAS*, *BRAF*, and *NRAS* alterations. Detailed characterization regarding the testing status for additional somatic variants in our LUAD case series is presented in Table S2. Although the number of rs78378222[C]-positive samples in the LUAD cohort (6/586) was small to perform meaningful comparisons, we were able to verify a similar mean and median age at tumor diagnosis between variant allele carriers and non-carriers’ groups (Table S2). 

**Table 2 -:** *TP53* rs78378222 (A>C) genotyping results.

Genotype	LUAD	ULM^a^	SARC
	N ( %)	N ( %)	N ( %)
	n=586	n=41	n=188
AA	580 (100)	41 (100)	188 (100)
AC	6 (1.02)	0	0
CC	0	0	0
C allele frequency	0.0051	0	0

^a^ Matched DNA samples obtained from tumor specimens and tumor-adjacent normal tissue (myometrium).

**Table 3 -:** Clinical and molecular findings from LUAD patients harboring the functional variant *TP53* rs78378222.

Identifier	Tumor/condition	Gender	Age at diagnosis (years)	Status of variants, LUAD^a^
#1	LUAD	Female	51	Negative
#2	LUAD	Male	80	Negative
#3	LUAD	Female	77	Positive; *KRAS* p.(Gly12Cys)^b^
#4	LUAD	Male	73	Positive; *KRAS* p.(Gly12Cys)^b^
#5	LUAD	Female	71	Negative
#6	LUAD	Female	44	Positive; *EGFR* p.(Met766_Ala767insAlaSerVal)^c^

^a^ LUAD cases were tested for somatic *EGFR*, *KRAS*, *BRAF* and *NRAS* variants. Specific regions evaluated in each gene were detailed in our previous study (PMID: 31532708).

^b^ Missense pathogenic variant located in the exon 2 of *KRAS* gene; it is an oncogenic (activating), clinically actionable *KRAS* alteration commonly identified in LUAD tumors.

^c^ Insertion located in the exon 19 of *EGFR* gene, representing a somatic oncogenic driver alteration in LUAD carcinogenesis.

Additional somatic sequence alterations in the *TP53* coding regions and exon-intron boundaries were detected by NGS in four variant-positive LUAD tumors (Table 4). In the other two positive LUAD cases, NGS analyses were not performed due to limitations in sample availability. The *TP53* positive tumors in NGS had at least one pathogenic/likely pathogenic alteration in the p53 DBD, of which three (75 %) exhibited the previously reported alteration *TP53* c.751A>C (p.Ile251Leu) with a low allele frequency (<0.05, expected for a somatic variant) in tumor DNA. LUAD tumors harboring this *TP53* exonic alteration and rs78378222[A/C] simultaneously did not exhibit either variations in age at diagnosis or other phenotypic features (data not shown).

**Table 4 -:** Additional *TP53* alterations identified by next generation sequencing analyses of the *TP53* entire coding region and exon-intron junctions (not including 3’UTR) in tumor DNA from four LUAD patients harboring the variant allele rs78378222[C].

Identifier^a^	WT^b^/mutant allele frequency	Coverage	ClinVar classification
#1	c.215C>G (0.56/0.44) c.672+62A>G (0.002/0.998) c.751A>C (0.96/0.04)	858x 990x 1,512x	Drug response^c^ Benign Pathogenic/Likely pathogenic^d^
#2	c.215C>G (0.47/0.53) c.672+62A>G (0.01/0.99) c.751A>C (0.97/0.03)	1,043x 1,013x 1,569x	Drug response^c^ Benign Pathogenic/Likely pathogenic^d^
#3	c.215C>G (0.96/0.04) c.524G>T (0.85/0.15) c.672+62A>G (0.04/0.96)	1,220x 717x 947x	Drug response^c^ Pathogenic/Likely pathogenic^e^ Benign
#4	c.215C>G (0.68/0.32) c.536A>T (0.63/0.37) c.672+62A>G (0.001/0.999) c.751A>C (0.96/0.04)	1,128x 744x 1,049x 2,000x	Drug response^c^ Uncertain significance^f^ Benign Pathogenic/Likely pathogenic^d^

^a^ NGS analyses were performed in only 4/6 positive LUAD cases due to limitations in sample availability for the remaining two patients, such as low DNA concentration and/or poor purity of tumor DNA.

^b^ WT, wild-type allele.

^c^ The common single nucleotide polymorphism *TP53* c.215C>G (p.Pro72Arg) (rs1042522, MAF=0.6) has been associated with response to antineoplastic agents (efficacy and toxicity) for certain tumor types, such as gastric, ovarian and breast cancers (reference: PMID 26696550).

^d^ The pathogenic/likely pathogenic variant *TP53* c.751A>C (p.Ile251Leu) was previously identified in one family with Li-Fraumeni syndrome (LFS, reference: PMID 21305319) and in a neoplasm of ovary (somatic origin, ClinVar database). This alteration is located in the DBD of p53 protein and is reported to have loss of transactivation capacity (reference: PMID 12826609).

^e^ The pathogenic/likely pathogenic variant *TP53* c.524G>T (p.Arg175Leu) has been observed in LFS patients (reference: PMID 16707427, ClinVar database). This alteration is in the DBD of p53 and showed a partial loss of transactivation activity and temperature sensitivity in functional assays (references: PMID 12826609, 14559903, 16861262). Another alteration at this same residue was associated with classic phenotype of LFS (reference: PMID 22233476, 21761402).

^f^ The variant of uncertain significance *TP53* c.536A>T (p.His179Leu) was previously detected in individuals with different tumor types (somatic origin in all cases; ClinVar database). This alteration has not been reported in the literature in individuals with *TP53*-related disease. It impairs p53 transactivation capacity, reduces apoptosis activity, confers cisplatin-sensitivity and increased cell mobility, and exhibits a dominant-negative effect (references: PMID 12826609, 16861262, 22114072, 23713777). Cells carrying this variant transplanted in mice induce tumor formation more rapidly than WT *TP53* cells (PMID: 9049183).

Importantly, homozygous individuals for the minor allele C were not identified in any of the study groups (Table 2). To contextualize this finding, we queried updated frequency data of this variant in population databases (gnomAD, 1000 Genomes, ExAC and ABraOM) and previous studies (Table S3). Given that it is a hypomorphic (i.e. multiple descriptions of p53 downregulation in clinical samples and cancer cell lines harboring the minor C-allele) and rare variant (MAF ranging from 0.002 to 0.02 in African and European populations, respectively), there are only a few reports of rs78378222[C/C] homozygotes in previous studies and different population databases (Table S3).

## Discussion

Several studies suggest that rare variants (MAF<0.05) have a more important functional consequence than common variants, and they tend to exhibit a stronger effect size than its counterparts (Gorlov *et al*., 2011). Therefore, rare variants are likely to be an essential element of the genetic basis of common human pathologies, including cancer (Bomba *et al*., 2017). The rare variant *TP53* 3’UTR rs78378222 (A>C) studied here was initially reported as a risk allele for diverse tumor types in European and North American populations (Stacey *et al*., 2011; Egan *et al*., 2012; Zhou *et al*., 2012; Enciso-Mora *et al*., 2013; Diskin *et al*. 2014; Wang *et al*. 2015). Later, it was described as a risk allele for development to several tumors, including brain cancers (predominantly glioma and neuroblastoma), esophageal squamous cell carcinoma, uterine leiomyoma, soft-tissue sarcoma, and non-melanomatous skin cancer mainly in cohorts of European ancestry (Melin *et al*. 2017; Rafnar *et al*., 2018; Deng *et al*., 2019; Di Giovannantonio *et al*., 2021). Moreover, Wang *et al.* (2016) conducted a meta-analysis that demonstrated the association of this variant with increased susceptibility to overall cancer.

Functional analyses have provided evidence supporting the wide-ranging association of this variant with both benign and malignant neoplasms. The *TP53* 3’UTR rs78378222 (A>C) variant changes the *TP53* PAS from AATAAA to AATACA, resulting in impaired *TP53* 3′-end processing, thereby decreasing p53 protein expression. In turn, the decreased p53 expression could affect other genes indirectly, through alterations in p53 downstream functions such as apoptosis (Stacey *et al*., 2011; Li *et al*., 2013; Macedo *et al*., 2016). A consistent finding among studies is the reduced levels of p53 in various human clinical specimens, cancer cell lines, and animal model tissues from individuals who carry the rs78378222[A/C] variant in a heterozygous state (Macedo *et al.*, 2016; Deng *et al*., 2019; Zhang *et al.*, 2021). Furthermore, the presence of the C nucleotide creates a binding site for miR-382-5p and compromises the miR-325-3p site, leading to p53 downregulation. The functional effect of this phenomenon has been demonstrated in a mouse model harboring the variant through miRNA expression analyses (Deng *et al.*, 2019). Additionally, another study reported that the C-allele introduces a miR-125b targeting site (Zhao *et al*., 2016). 

Herein, we identified a low prevalence of rs78378222[C] carriers in LUAD from Southern Brazil (1.02 %), as well as absence of this variant in a small cohort of ULM-affected women and sarcomas from the same population. The low prevalence of rs78378222[C] carriers observed in each of the cohorts and in the overall sample of the current study (6/815, 0.7 %) are similar to the frequency recently reported in a Brazilian repository of whole-genome sequencing data (1.2 %,14/1,171) from unrelated and healthy elderly individuals from general population of São Paulo, Brazil (ABraOM, SABE-WGS-1171 dataset) (Naslavsky *et al*., 2017; Naslavsky *et al.*, 2020) and with our previous findings in population controls (1 %) (Macedo *et al*., 2016).

In the scientific literature, there is only one previous study that evaluated rs78378222[C] in lung cancer cases (not specified histological classification) (Guan *et al*., 2013). They found a higher carrier frequency of 21/1013 (2,12 %) among non-Hispanic caucasians from the USA population diagnosed with this tumor type. However, they did not find an association of the rs78378222[C] allele with lung cancer risk and, unlike our study, they genotyped germline samples. A high germline prevalence of carriers (~2 %) has also been previously reported in a robust meta-analysis of two GWAS involving ULM-affected women from Iceland and UK (n = 16,595 cases). They found a significant association between the variant and ULM in these populations (OR = 1.74, 95 % CI = 1.6 to 1.89) (Rafnar *et al*., 2018). Moreover, Deng *et al*. (2019) found a significant association between rs78378222[C] and the risk in soft tissue sarcoma (OR = 3.29, *P*= 0.0014) in a germline context. In the past years, a limited number of studies have explored the somatic occurrence of this variant in other tumor types (Li *et al*., 2013; Wang *et al*., 2016; Voropaeva *et al*., 2020; Zhang *et al.*, 2021). Importantly, our study described, for the first time, the low somatic prevalence of minor C-allele in LUAD cases. Besides that, 3/6 (50 %) of rs78378222 heterozygous LUAD tumors exhibited co-occurrence of somatic driver gene variants (*KRAS* or *EGFR*). Together, these data suggest that the rs78378222 variant does not play a determining role in LUAD occurrence in the population of Southern Brazil.

Other interesting findings from the present study can be highlighted. First, we explored the presence of other somatic *TP53* functional/pathogenic variants in lung cancer specimens from rs78378222[C] carriers by NGS analyses, which allowed us to identify that all four variant-positive tumors with sample availability had additional pathogenic or likely pathogenic variants in the *TP53* coding regions. Most of the LUAD tumors (3/4, 75 %) had the same pathogenic/likely pathogenic sequence variant, namely *TP53* c.751A>C (p.Ile251Leu), located in the p53 DBD and reported to cause loss of transactivation capacity (Kato *et al*., 2003). It was previously detected in one Li-Fraumeni Syndrome (LFS) family (Wu *et al*., 2011) and in a neoplasm of ovary (ClinVar database, 2021) in germline and somatic context, respectively. In contrast with our findings in LUAD tumors, recent studies have identified that, regardless of tumor type, rs78378222[C] is more frequent in tumors with no somatic-coding pathogenic *TP53* variants, i.e. WT *TP53* tumors (Voropaeva *et al*., 2020; Zhang *et al.*, 2021). The rs78378222[C] minor allele was not associated with lung cancer (not specified distribution of histological types) in a case series from the USA (Guan *et al*., 2013), 

The results of the current study must be interpreted in the context of the following limitations: (1) although our overall sample size is more than 800 individuals and a recent study in the Russian population described a very high somatic frequency of rs78378222[C] carriers analyzing only 136 cases of a lymphoma subtype (11/136, 8.1 %) (Voropaeva *et al*., 2020), our cancer cohorts actually have a relatively small number of patients for analysis of a rare variant in ULM (n = 41) and SARC (n = 188) study groups; (2) LUAD tumor samples were obtained from a retrospective study and de-identified for use in the current study, hindering the complete clinical characterization of this case series regarding the ethnic ancestry, cancer family history, and histological subtype; (3) the other two positive LUAD cases whose NGS analyses were not performed due to limitations in sample availability could be important for more robust interpretations of LUAD samples with *TP53* variants co-occurring with rs78378222[C].

In conclusion, when compared to previous studies from different populations, the prevalence of *TP53* rs78378222[C] carriers in our case series from Southern Brazil (1,05 % in LUAD cases) is similar to that observed in the admixed-general population of the country (~1 %). This is the largest study in somatic tumors evaluating a noncoding *TP53* functional variant in Brazilians. Additionally, our study examined, for the first time, the somatic frequency of the *TP53* 3’UTR variant in lung cancer specimens with known histological classification (LUAD). Overall, our findings suggest that further analyses genotyping the rs78378222 variant would not be informative for LUAD, SARC and ULM tumor types in that studied Brazilian regions. However, different types of sporadic tumors should be evaluated to determine if the screening for this alteration is justified in the cancer-affected Brazilian patients.

## Supplementary material

The following online material is available for this article:

Table S1 - Specific clinical features according to the study groups (tumor types).

Table S2 - Comparison of clinical and molecular features between LUAD samples of *TP53* rs78378222[C] variant allele carriers and non-carriers.

Table S3 - Frequency of the *TP53* rs78378222[C] variant allele reported in previous studies and population databases.
