# Supplementary material for: Exploring the frequency of a TP53 polyadenylation signal variant in tumor DNA from patients diagnosed with lung adenocarcinomas, sarcomas and uterine leiomyomas
Source: Genet Mol Biol. 2024 Jan 19;46(3 Suppl 1):e20230133. doi: 10.1590/1678-4685-GMB-2023-0133 (PMC10802224; doi:10.1590/1678-4685-GMB-2023-0133)
Supplement: Table S1 - [file 1415-4757-GMB-46-03-s1-e20230133-s1.pdf]

Genetics and Molecular Biology
https://doi.org/10.1590/1678-4685-GMB-2023-0133

Supplementary Material to "Exploring the frequency of a *TP53* polyadenylation signal variant in tumor DNA from patients diagnosed with lung adenocarcinomas, sarcomas and uterine leiomyomas"

Table S1 - Specific clinical features according to the study groups (tumor types).

Clinical data	Lung adenocarcinoma, LUAD (n=586)	Uterine leiomyoma, ULM (n=41)	Sarcoma, SARC (n=188)
Histological subtype, N (%)	72 (12.3)[a,b]	NA	NA
Acinar	47 (65.3)	NA	NA
Lepidic	25 (34.7)	NA	NA
Uterine location, N (%)		33 (80.5)[a,c]	
Intramural	NA	17 (51.5)	NA
Subserosal	NA	1 (3.1)	NA
Intramural/Subserosal	NA	8 (24.2)	NA
Intramural/Subserosal/ Submucosal	NA	7 (21.2)	NA
Sarcoma origin tissue			188 (100)
Bone	NA	NA	57 (30.3)
Soft Tissue	NA	NA	130 (69.2)
Other [d]	NA	NA	1 (0.5)

NA, not applicable.
[a] The percentage was calculated over the total number of genotyped samples for each study group, and over the number of cases for which the specified clinical data was available.
[b] Histological subtype data were available for only a small proportion (72/586) of LUAD patients.
[c] Uterine location for the ULM's group was available for the most (33/41) affected women.
[d] This tumor represents 1 case of leiomyosarcoma.
