# Supplementary material for: Exploring the frequency of a TP53 polyadenylation signal variant in tumor DNA from patients diagnosed with lung adenocarcinomas, sarcomas and uterine leiomyomas
Source: Genet Mol Biol. 2024 Jan 19;46(3 Suppl 1):e20230133. doi: 10.1590/1678-4685-GMB-2023-0133 (PMC10802224; doi:10.1590/1678-4685-GMB-2023-0133)
Supplement: Table S2 - [file 1415-4757-GMB-46-03-s1-e20230133-s2.pdf]

Genetics and Molecular Biology
https://doi.org/10.1590/1678-4685-GMB-2023-0133

Supplementary Material to "Exploring the frequency of a *TP53* polyadenylation signal variant in tumor DNA from patients diagnosed with lung adenocarcinomas, sarcomas and uterine leiomyomas"

Table S2 - Comparison of clinical and molecular features between LUAD samples of *TP53* rs78378222[C] variant allele carriers and non-carriers.

**Features**[a]	**All samples** n=586	**Carriers** n=6	**Non-carriers** n=580
Age at diagnosis, N (%)[a]	586 (100)[a]		
Mean, years (±SD)	66.1 (11.9)	66 (14.8)	66.1 (11.9)
Median, years (IR)	67 (16)	72 (20)	66 (16)
Patients tested for alterations, N (%)[a,b]	502 (85.7)[a,b]	6 (100)	496 (85.5)
EGFR variant	106 (21.1)	1 (16.7)	105 (21.2)
KRAS variant	148 (29.5)	2 (33.3)	146 (29.4)
BRAF variant	15 (3)	0	15 (3)
None	233 (46.4)	3 (50)	230 (46.4)

SD, standard deviation; IR, interquartile range.

[a] The percentage was calculated over the total number of genotyped samples (586) and over the number of cases for which the specified clinical or molecular data was available.

[b] A total of 502/586 LUAD cases were tested for somatic *EGFR*, *KRAS*, *BRAF*, and *NRAS* variants. Specific regions evaluated in each gene are detailed in our previous study (PMID: 31532708). *NRAS* somatic variants were not found in our case series.
