# Supplementary material for: Exploring the frequency of a TP53 polyadenylation signal variant in tumor DNA from patients diagnosed with lung adenocarcinomas, sarcomas and uterine leiomyomas
Source: Genet Mol Biol. 2024 Jan 19;46(3 Suppl 1):e20230133. doi: 10.1590/1678-4685-GMB-2023-0133 (PMC10802224; doi:10.1590/1678-4685-GMB-2023-0133)
Supplement: Table S3 - [file 1415-4757-GMB-46-03-s1-e20230133-s3.pdf]

Genetics and Molecular Biology
https://doi.org/10.1590/1678-4685-GMB-2023-0133

Supplementary Material to "Exploring the frequency of a *TP53* polyadenylation signal variant in tumor DNA from patients diagnosed with lung adenocarcinomas, sarcomas and uterine leiomyomas"

Table S3 - Frequency of the *TP53* rs78378222[C] variant allele reported in previous studies and population databases.

Source/Population database or previous study	*TP53* rs78378222 (A>C)		
	N[a]	Homoz.[b]	MAF[c]
gnomAD (Overall population)	15,690	4	0.012
1000 Genomes	2,504	0	0.0026
ExAC (Overall and specific populations)	NR[d]	NR[d]	NR
ABraOM (Online Archive of Brazilian Mutations)	1,171	0	**0.0059**
Cancer-unaffected Brazilian women (Macedo *et al.*, 2016)[e]	299	0	**0.005**
Lung cancer cases in the USA (histological subtype not specified) (Guan *et al.*, 2013)	1013	0	0.01
European uterine leiomyoma cases (Rafnar *et al.*, 2018)	16,595	NR[d]	0.018
Chinese sarcoma cases (Deng *et al.*, 2019)	130	NR[d]	0.0538

[a] N, sample number evaluated in the specific database and/or study.
[b] Homoz., number of variant allele homozygotes.
[c] MAF, minor allele frequency (frequency of variant allele); MAF in Brazilian and/or Latino populations is highlighted in bold letters.
[d] NR, not reported.
[e] Control group in our previous study (PMID: 26823150) was composed of cancer-unaffected women from the general population in southern Brazil, presenting no clinical evidence and/or suspicion of breast cancer, and their family histories were not consistent with hereditary cancer syndromes
